# Influence of lengths of millimeter-scale single-walled carbon nanotube on electrical and mechanical properties of buckypaper

**DOI:** 10.1186/1556-276X-8-546

**Published:** 2013-12-27

**Authors:** Shunsuke Sakurai, Fuminori Kamada, Don N Futaba, Motoo Yumura, Kenji Hata

**Affiliations:** 1 Technology Research Association for Single Wall Carbon Nanotubes (TASC), Central 5, 1-1-1 Higashi, Tsukuba, Ibaraki 305-8565, Japan; 2 National Institute of Advanced Industrial Science and Technology (AIST), Central 5, 1-1-1, Higashi, Tsukuba, Ibaraki 305-8565, Japan; 3 Japan Science and Technology Agency (JST), Honcho 4-1-8, Kawaguchi 332-0012, Japan

**Keywords:** Carbon nanotube, Buckypaper, Tube length

## Abstract

The electrical conductivity and mechanical strength of carbon nanotube (CNT) buckypaper comprised of millimeter-scale long single-walled CNT (SWCNT) was markedly improved by the use of longer SWCNTs. A series of buckypapers, fabricated from SWCNT forests of varying heights (350, 700, 1,500 μm), showed that both the electrical conductivity (19 to 45 S/cm) and tensile strength (27 to 52 MPa) doubled. These improvements were due to improved transfer of electron and load through a reduced number of junctions for longer SWCNTs. Interestingly, no effects of forest height on the thermal diffusivity of SWCNT buckypapers were observed. Further, these findings provide evidence that the actual SWCNT length in forests is similar to the height.

## Background

The effective transfer of phonons, electrons, and load is known to increase with longer carbon nanotubes (CNTs) within CNT agglomerates. For example, in the percolation theory, electron transfer is expected to be achieved with a lesser number of CNTs by the use of longer CNTs in accordance with the relation *N*_c_ = 5.71 /*L*_s_^2^, where *N*_c_ and *L*_s_ are percolation threshold and CNT length, respectively [1-4]. For example, higher electrical conductivity was observed for transparent conductive films using network thin films of longer CNTs [5,6]. In addition, Miyata el al. reported a field effect transistor (FET) with high mobility using long single-walled CNTs (SWCNTs) [7]. Further, in CNT/polymer composites, the beneficial effect of CNT length on the efficiency of phonon/electron transport and interfacial load transfer has been reported [8-11]. Such superiority in properties from long CNTs originates from the fewer CNT junctions, which interrupt phonon, electron, and load transfer, in a network structure of CNTs required to span the material.

Although these reports suggest the advantages of long CNTs on electron, thermal, and mechanical properties of a CNT assembly, this point has not been explicitly demonstrated experimentally. In other words, almost all the above experiments have employed only short CNTs, on the order of micrometers, with only one exceptional report by Zhu et al., who reported on the properties of composite of multiwalled CNTs with thick diameters (approximately 40 to 70 nm) and bismaleimide (BMI) [8]. Particularly, there has been no report on the effect of length on the properties of SWCNTs exceeding 1 mm.

There are three reasons why research on the CNT length dependence of various properties of CNT assemblies has been difficult. First, the synthesis of long CNTs with uniform length in a large quantity is difficult. For example, Wang et al. reported the synthesis of long single-wall CNTs with a maximum length of 18.5 cm, but there were substantial variations in CNT length [12]. Cao et al. reported an interesting approach for length-tunable CNT growth, but the length did not reach to millimeter scale [13]. Furthermore, several groups reported the methods for classifying long/short CNTs, but this was not applied to CNTs that were longer than 10 μm in length [14-17]. Secondly, due to the tight entanglement among CNTs, the dispersion of CNTs without CNT scission is difficult. Ultrasonic agitation, which has been typically employed as a dispersion method, is known to shorten CNTs as it disentangles them [18]. Finally, there is no available method to measure the lengths of individual CNTs longer than 100 μm. CNTs with lengths of several micrometers have been evaluated by atomic force microscopy (AFM) [8-11,14-17], but this method encounters extreme difficultly when obtaining statistically significant data for long CNTs.

Using water-assisted chemical vapor deposition (CVD), we reported the synthesis of a vertically aligned SWCNT array (SWCNT forest) with height exceeding a millimeter [19]. The SWCNT forests possessed several excellent structural properties, such as long length, high purity, and high specific surface area. This development opened up the potential for various new applications of CNTs, such as high-performance super-capacitors [20-23] and highly durable conductive rubbers [24,25]. Subsequently, many groups reported the growth of long SWCNTs. For example, Zhong et al. reported the growth of SWCNT forests reaching 0.5 cm in length [26]. Hasegawa et al. reported growth of SWCNT forests of several millimeters in length without an etching agent (water) [27]. Numerous studies have also reported the synthesis of multiwalled CNT forests [28-30]. However, the following points remain unclear at present: the correlations between forest height and (1) the actual CNT length and (2) the electrical, thermal, and mechanical properties after formation of CNT assemblies.

In this research, we report the effect of the length of long CNTs on the electrical, thermal, and mechanical properties. Our results demonstrated a strong dependence of the SWCNT aggregate properties on the length. Specifically, buckypaper produced from 1,500 μm SWCNT forests exhibited approximately twice the electrical conductivity (52 vs. 27 S/m) and twice the tensile strength (45 vs. 19 MPa) of a buckypaper produced using 350 μm SWCNT forests. The use of an automated synthetic system equipped with height monitoring and dispersion strategy recently reported by Kobashi et al. [31] allowed overcoming the first two of the aforementioned issues, namely the required large quantity of long CNTs and CNT dispersion method to preserve length.

## Methods

### Fabrication of uniform buckypaper from SWCNTs of varying length

**Figure 1:**
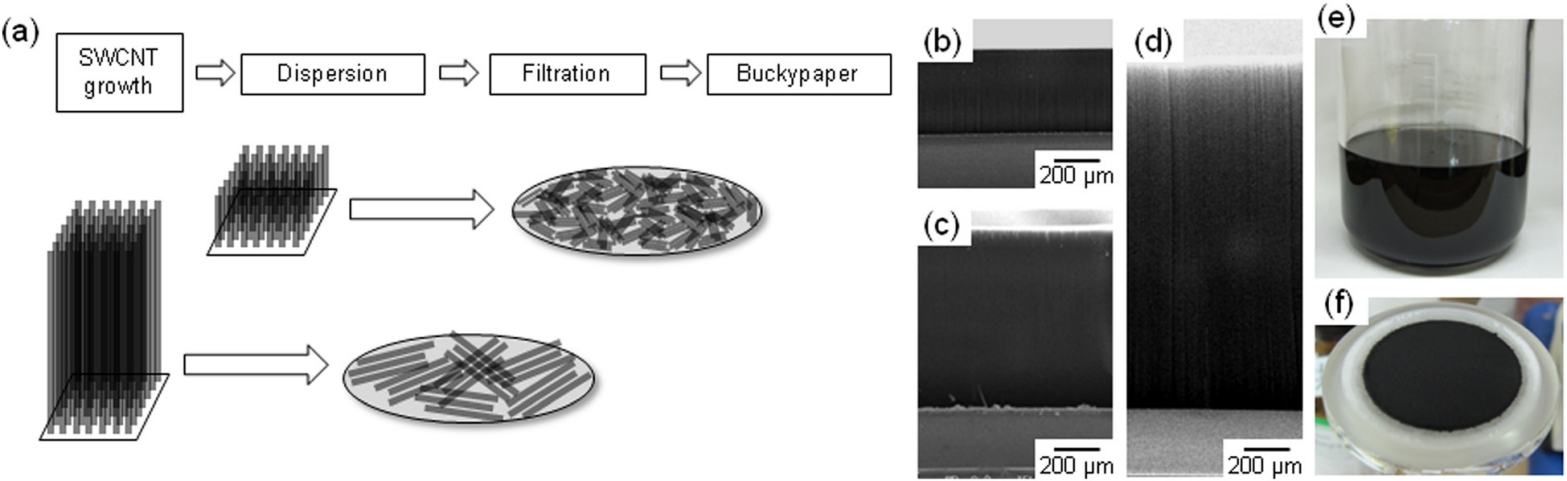
**Schematic representation of fabrication process, SEM images of SWCNT forest, photographs of buckypaper and of dispersion of SWCNT. (a)** Schematic representation of the fabrication process of buckypaper comprising SWCNT forest with different heights. SEM images of SWCNT forest with **(b)** 350-, **(c)** 700-, and **(d)** 1,500-μm heights. **(e)** Photograph of the dispersion of SWNCT. **(f)** Photograph of the buckypaper obtained after the filtration.

We selected the buckypaper, a randomly oriented sheet of CNT, as the form of CNT assembly to study the effect of SWCNT length. The buckypaper is particularly suitable for the present study because it is comprised solely of CNTs (i.e., no binder or other foreign material), and the fabrication is relatively simple, merely requiring filtration of a SWCNT dispersion. We fabricated a series of buckypapers from SWCNT forests of different heights, which are schematically illustrated in Figure 1a. The fabrication process comprises three main steps: (1) synthesis of SWCNT forests of determined length; (2) dispersion of the SWCNTs; and (3) fabrication of the buckypaper.

SWCNT forests of various lengths were synthesized in a fully automated CVD synthetic system equipped with a telecentric height measurement system using the water-assisted CVD process. A Fe/Al_2_O_3_ catalyst-sputtered silicon substrate was inserted into the 1-in. diameter quartz tube reactor (1 atm, 750°C). First, the substrate was exposed to a carrier gas (He, total flow of 1,000 sccm) containing hydrogen (40%) to form catalytic nanoparticles, and then SWCNTs were synthesized using a C_2_H_4_ (100 sccm) carbon feedstock and precisely regulated water vapor (100 to 150 ppm). The SWCNT forest height was controlled by using the height as feedback to the control software to automatically stop when the target height was achieved [32]. In this way, SWCNT forests with precisely regulated heights (350, 700, 1,500 μm) could be synthesized in mass quantities. The uniformity of SWCNT forest heights was verified by scanning electron microscopy (SEM; Figure 1b,c,d) and digital photography (see Additional file 1: Figure S1).

Next, dispersions of the series of SWCNT forests of differing heights were prepared. Although conventional dispersion strategies aim to completely disentangle the CNTs into isolated particles, it also results in scission. Our strategy minimizes the scission by suspending the SWCNT agglomerates in a solvent while retaining the entanglement (Yoon et al.: Controlling the balance between exfoliation and damage during dispersion long SWCNTs for advanced composites, unpublished). We selected jet milling as the dispersion method because it has shown to preserve the SWCNT length with minimal scission, and it has also been shown that the resulting materials are suitable to fabricate SWCNT/polymer composite materials of high electrical conductivity (Yoon et al.: Controlling the balance between exfoliation and damage during dispersion long SWCNTs for advanced composites, unpublished) [24,25,33]. This benefit stems from a turbulent flow mechanism used in jet milling which exfoliates CNTs with minimal damage, in contrast to the cavitation mechanism used in conventional ultrasonic dispersion which is known to damage CNTs [33]. Mixtures of SWCNT forest samples of specific length in methyl isobutyl ketone (MIBK) were introduced into a high-pressure jet-milling homogenizer (Nano Jet Pal, JN10, Jokoh), and suspensions (0.03 wt.%) were made by a high-pressure ejection through a nozzle (20 to 120 MPa, single pass).

Finally, a series of buckypapers with precisely controlled mass densities were prepared by the filtration and compression processes described below. The suspensions were carefully filtered using metal mesh (500 mesh, diameter of wire 16 μm). The as-dried buckypapers (diameter 47 mm) were removed from the filters and dried under vacuum at 60°C for 1 day under the pressure from 1-kg weight. Some papers were further pressed into a higher density in order to eliminate the effects of mass density on buckypaper properties. Although the mass densities of the as-dried buckypaper significantly varied among the samples (0.25 to 0.44 g/cm^3^, Table 1), buckypapers with uniform density, regardless of forest height, were obtained by pressing buckypapers at 20 and 100 MPa to raise the density at approximately 0.50 g/cm^3^ (0.48 to 0.50 g/cm^3^) and 0.63 g/cm^3^ (0.61 to 0.65 g cm^**–**3^), respectively (Table 1). In addition, buckypaper samples were uniform where the thicknesses at its periphery and at the middle were nearly identical.

**Table 1:** The average thickness and mass densities of buckypapers prepared from SWCNT forest with different height

**Height of SWCNT forest (μm)**	**Buckypaper**	**Average thickness (μm)**	**Mass density (g/cm**^**3**^**)**
350	As-dried	72	0.40
	As-dried	62	0.37
	Compressed at 20 MPa	46	0.50
	Compressed at 100 MPa	41	0.61
700 μm	As-dried	58	0.44
	As-dried	73	0.33
	Compressed at 20 MPa	47	0.48
	Compressed at 100 MPa	39	0.62
1500 μm	As-dried	73	0.32
	As-dried	92	0.25
	Compressed at 20 MPa	49	0.50
	Compressed at 100 MPa	38	0.65

For each height of SWCNT forest, two as-dried buckypapers, one paper after compression at 20 MPa, and one paper after compression at 100 MPa have been prepared. The thickness of the buckypaper was measured by the stylus method instrument. The average thickness of five measurements was obtained from both of the center and the edge of buckypapers.

## Results and discussions

### High electrical conductivity in buckypaper fabricated from high SWCNT forests

**Figure 2:**
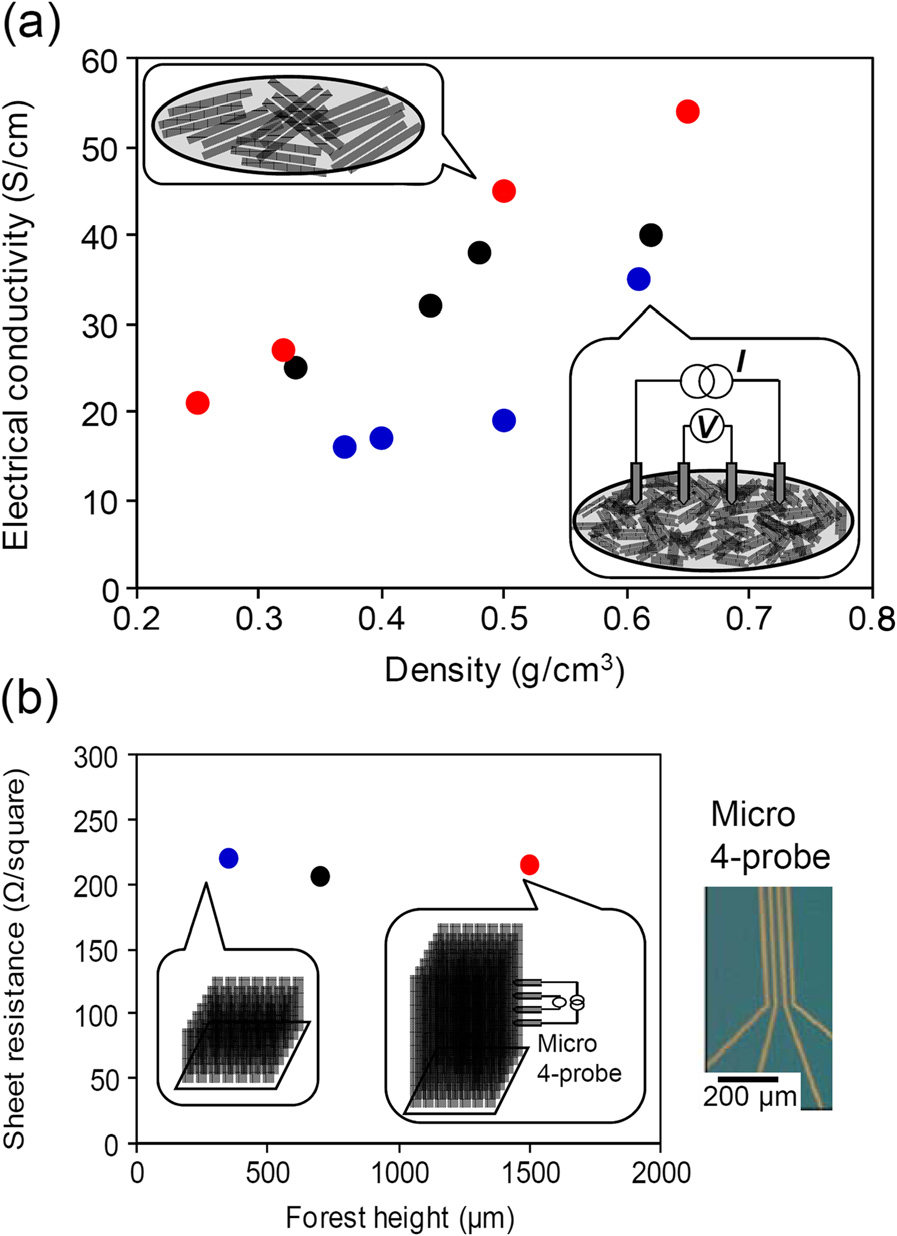
**Electrical conductivity of buckypapers (a) and sheet resistance of SWCNT forest (b). (a)** The electrical conductivity of buckypapers as a function of the mass density of buckypapers. Red, black, and blue dots indicate the buckypaper fabricated from SWCNT forest with the heights of 1,500, 700, and 350 μm, respectively. **(b)** Sheet resistance of SWCNT forest with different heights measured by a micro 4-probe. Red, black, and blue dots indicate the SWCNT forest with the heights of 1,500, 700, and 350 μm, respectively. Inset shows the photograph of the gold electrode on Si substrate used as a micro 4-probe.

We found that buckypaper fabricated from tall SWCNT forests exhibited excellent electrical conductivity and mechanical strength. In terms of electrical properties, the electrical conductivity (*σ*) of each buckypaper sample was calculated by *σ* = 1/*tR*_s_ (*t* = average buckypaper thickness) from the sheet resistance (*R*_s_) measured using a commercially available four-probe resistance measuring apparatus (Loresta-GP, Mitsubishi Chemical Analytech Co., Ltd., Yokohama, Japan) The electrical conductivity of buckypapers made from forests of the same height exhibited a linear dependence on density (Figure 2a). For example, the electrical conductivity rose from 21 to 54 S/cm with a density increase from 0.25 to 0.65 g/cm^3^. Significantly, we observed that the taller the forest used in the buckypaper fabrication, the higher the electrical conductivity. Comparing buckypapers with almost the same density, the buckypaper obtained from forests with heights of 1,500 μm exhibited approximately twice the electrical conductivity of buckypaper made from 350-μm forests, (i.e., 45 vs. 19 S/cm at 0.50 g/cm^3^, and 27 vs. 16 S/cm around 0.35 g/cm^3^).

In order to verify that this apparent height-dependent variation in buckypaper conductivity was not due to differences in CNT quality, which has been shown to be essential for the various properties of buckypaper in previous works [34], Raman spectroscopy and electrical resistivity measurements of the as-grown SWCNT forests were carried out. The intensity ratios of the G-band (1,600/cm) and the D-band (1,350/cm) in the Raman spectra (see additional file 1: Figure S2), an indicator of CNT quality, were very similar (approximately 7). Peak positions and intensities in the radial breathing modes (RBM; 100 to 300/cm) were also nearly identical for all SWCNT forest heights. As the RBM peak position *w* (cm^-1^) is reported to be inversely proportional to the SWCNT diameter (nm), i.e., *w* = 248/*d*[35], these findings indicate that the effect of forest height on SWCNT diameter distribution was small. Furthermore, electrical conductivity of raw material forest was evaluated by applying a micro 4-probe onto the sides of SWCNT forests. Since the distances between the probes (50 μm) in a micro 4-probe was sufficiently short compared with the forest height, CNT length had almost no influence on the resistance values observed with this measurement. The measured resistance was nearly identical (206 to 220 Ω/sq) regardless of forest height (Figure 2b), indicating that quality of the SWCNTs did not degrade when growing forests of height to 1,500 μm, in accordance with the results of Raman spectroscopy.

As shown in the previous paragraph, taking into consideration the fact that forest height did not influence CNT quality, we conclude that the increase in buckypaper conductivity accompanying forest height was a result of the increased length of individual SWCNTs. In other words, improved electron transfer, thus higher conductivity, became possible from fewer junctions as a result of individual SWCNTs becoming longer. Furthermore, the 50% drop in buckypaper resistance by the approximately fourfold increase in SWCNT length (350 to 1,500 μm in forest height) indicate the strong effect of CNT-CNT junctions on the electrical resistance of SWCNT assemblies.

### High tensile strength in buckypaper fabricated from high SWCNT forests

**Figure 3:**
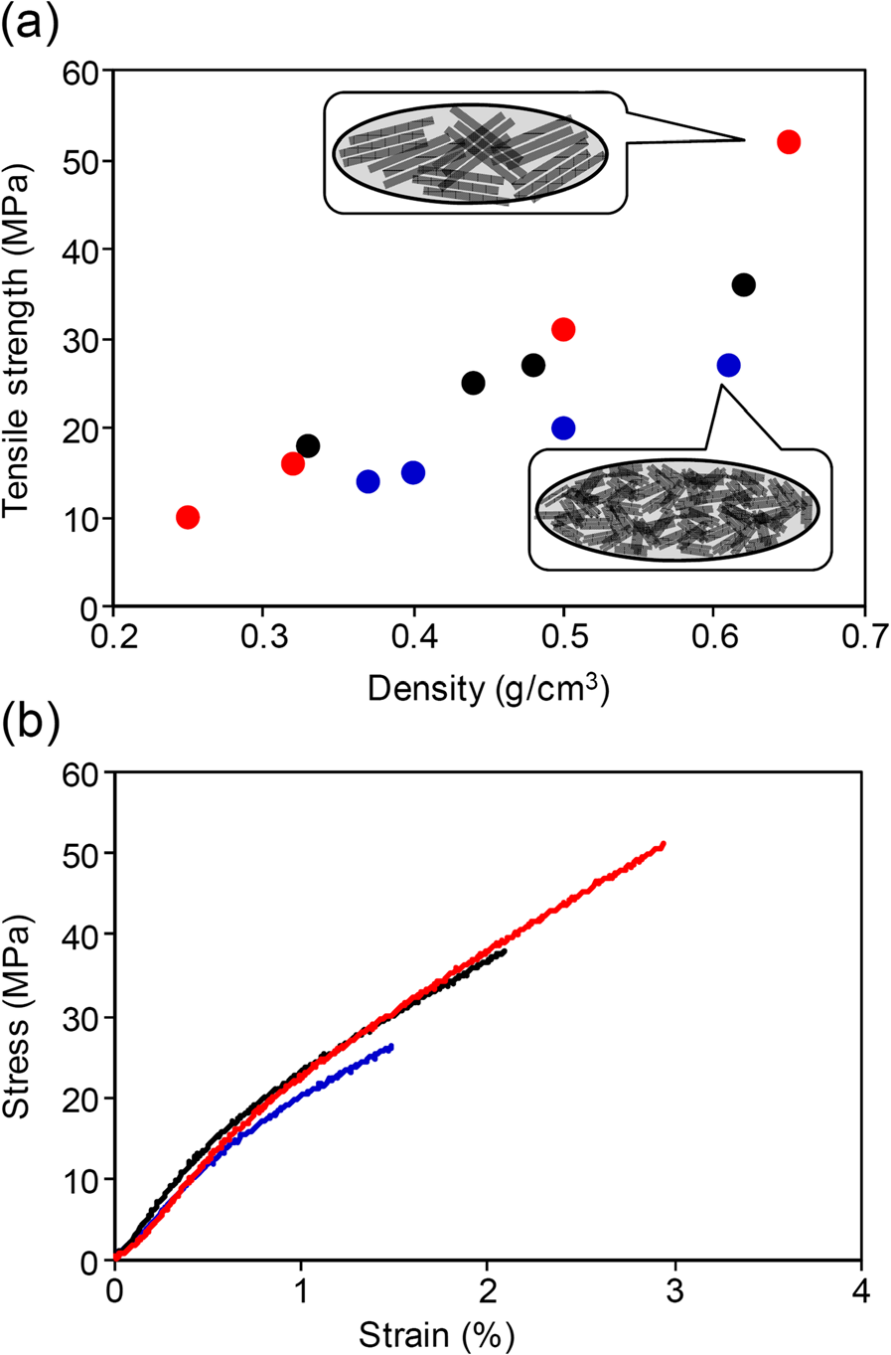
**Tensile strength (a) and stress–strain curves of buckypapers (b). (a)** The tensile strength of buckypapers as a function of the mass density of buckypapers. **(b)** Red, black, and blue dots indicate the buckypaper fabricated from SWCNT forest with the heights of 1,500, 700, and 350 μm, respectively.

Another advantage of buckypaper made from tall SWCNT forests shown by the present study for the first time is the improved mechanical properties, i.e., high tensile strength and breaking strain. Tensile test samples were cut into a dog bone-shape from the sheet with the dimension of 40 mm (length) × 2 mm (width). The extension rate and the gauge length were 1.0 mm/min and 20 mm, respectively. The tests were performed using a Micro Autograph MST-I (Shimadzu Co., Kyoto, Japan) with 100-N load cell. As reported by previous papers [34], tensile strength increased linearly with the mass density (Figure 3a); therefore, we compared the mechanical properties of buckypapers of similar mass densities approximately 0.63 g/cm^3^. Importantly, for an increase in forest height from 350 to 1,500 μm, both tensile strength and breaking strain increased by about 100% (27 to 52 MPa and 1.5% to 2.9%, respectively). In other words, the use of taller forests resulted in buckypapers which could withstand larger loads and strains. There were no major differences in Young’s modulus (i.e., stress/strain) regardless of forest height indicating similar interfacial contact between CNTs, as shown in Figure 3b. The mechanism by which mechanical strength was observed to improve through using tall forests can be interpreted in an analogous manner to that for improvement in electrical conductivity; in other words, the longer the CNT, the fewer the junctions as weak points for load transfer.

### Relationship between forest height and SWCNT length

Additional insight can be garnered from the improvement in electrical and mechanical properties in tall forests on the actual length of the SWCNTs in a forest. Thus far, no direct evidence has been shown regarding this point. Our results indicate that the length of the SWCNTs within the forest is equal to the forest height.

Furthermore, we quantitatively discuss the effect of individual SWCNT length on electrical conductance and load transfer. Assuming that the electrical and mechanical properties are related to the number of junctions, the approximate double electrical conductivity and tensile strength exhibited by forests with heights of 1,500 μm compared with those of heights 350 μm indicates that half the number of SWCNT junctions are present for electron/load. We estimated that the SWCNTs from a 1,500-μm forest were, in fact, four times longer than those in a 350-μm forests by constructing a simple model describing the effective area of a SWCNT of a certain length as it spreads in a buckypaper. To make this model solvable, we assumed that the SWCNTs fell into a circular island with a uniform areal mass (i.e., SWCNT mass per unit area) within the buckypaper plane. The uniform areal mass assumption is justified by the overall macroscopic homogeneity of the buckypaper. With this consideration, the diameter of the effective area is proportional to the square root of the SWCNT length, and the effective area, where a SWCNT can make contact with another effective area, would be proportional to the length of the SWCNT. Therefore, we find that the four-time difference in forest height (1,500:350) matches well with the four-time difference in effective areas which would result in a twofold difference in junctions along a path and thusly explain the difference in electrical conductivity and mechanical strain. Importantly, we can also conclude that the length of a SWCNT within a forest, at least to a large extent, spans the height of the forest from the substrate to the forest top.

### Relationship between buckypaper thermal conductivity and high SWCNT forest height

**Figure 4:**
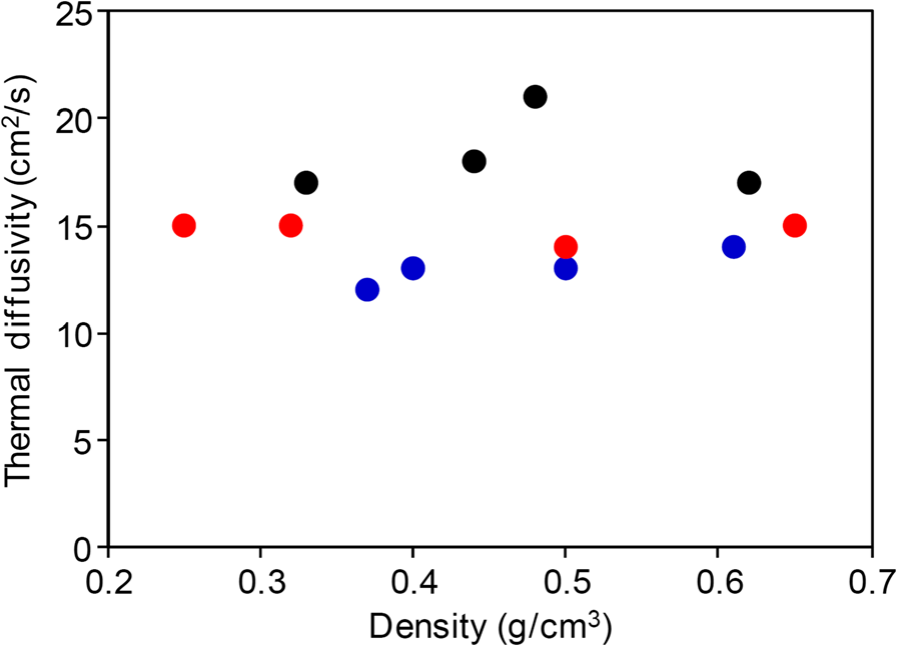
**Thermal diffusivity of buckypapers in horizontal direction as a function of mass density of buckypapers.** Red, black, and blue dots indicate the buckypaper fabricated from SWCNT forest with the heights of 1,500, 700, and 350 μm, respectively.

Furthermore, we investigated the in-plane thermal diffusivities of buckypaper fabricated from SWCNT forests of various heights. Thermal diffusivities of buckypaper in horizontal direction were measured by the Thermowave Analyzer (Bethel Co., Ibaraki, Japan) at room temperature. As opposed to electrical conductivity, a clear dependence of thermal conductance on SWCNT forest height was not observed (Figure 4). In particular, the tallest forests (1,500 μm) did not exhibit the highest thermal diffusivity (15 cm^2^/s), while forest with a medium height of 700 μm showed a slightly higher thermal diffusivity (18 cm^2^/s). These findings can be explained by theoretical prediction [33] and our recent experimental results that the thermal diffusivity of SWCNT forests is strongly dependent on the crystallinity (or the G-band/D-band ratio) [36]; in other words, while junctions between SWCNTs play the rate-limiting factor in electrical conductivity, phonon scattering via defects in individual SWCNTs appears dominant for thermal diffusivity. The number of junctions appears to only exhibit a small influence. This fact indicates that highly crystalline CNTs, not length, is most important for creating CNT networks with superior thermal conductivity.

## Conclusions

The present research shows that long SWCNTs with lengths exceeding the millimeter order greatly improved the electrical conductivity and mechanical strength of buckypaper. We attribute these improvements to electron and load transfer being improved through a reduced number of junctions due to increased CNT length. In addition, we conclude that the lengths of SWCNTs in forests that attain heights of 1,500 μm were close to that of the forest height. These findings indicate the need for taller SWCNT forests in the fabrication of buckypaper for high electrical conductivity and mechanical strength. Recently, Di et al. reported the ultrastrong and highly conducting CNT film by direct drawing from spinnable CNT array, where the tube length is around 220 μm [34]. Our finding in this study suggest the possibility that the properties of CNT directly drawn from CNT forest can be further enhanced by using longer CNT array. In addition, we expect that using tall SWCNT forests would also raise the conductivity and mechanical strength of SWCNT networks in SWCNT/polymer composite materials.

## Competing interests

The authors declare that they have no competing interests.

## Authors’ contributions

SS and KH designed the experiments. SS, FK, and DNF conducted CNT synthesis. FK conducted fabrication and characterization of buckypaper. SS and KH prepared the manuscript. All authors read and approved the final manuscript.

## Supplementary Material

Additional file 1 **Photograph and Raman spectra of SWCNT forest with different heights. Figure S1.** Photograph of SWCNT forest with different heights with Si substrate. **Figure S2.** Raman spectra of SWCNT forest with different heights (excitation wavelength 532 nm). Click here for file
