# Supplementary material for: Influence of lengths of millimeter-scale single-walled carbon nanotube on electrical and mechanical properties of buckypaper
Source: Nanoscale Res Lett. 2013 Dec 27;8(1):546. doi: 10.1186/1556-276X-8-546 (PMC3884011; doi:10.1186/1556-276X-8-546)
Supplement: Additional file 1 — Photograph and Raman spectra of SWCNT forest with different heights. Figure S1. Photograph of SWCNT forest with different heights with Si substrate. Figure S2. Raman spectra of SWCNT forest with different heights (excitation wavelength 532 nm). [file 1556-276X-8-546-S1.pdf]

Supporting information

Influence of lengths of mm-scale single-walled carbon nanotube on electrical and mechanical properties of buckypaper

Shunsuke Sakurai[1,2], *Fuminori Kamada*[1], *Don Futaba*[1,2], *Motoo Yumura*[1,2], *and Kenji Hata*[1,2,3,]*

[1] Technology Research Association for Single Wall Carbon Nanotubes (TASC), Central 5, 1-1-1 Higashi, Tsukuba, Ibaraki 305-8565, Japan

[2] National Institute of Advanced Industrial Science and Technology (AIST), Central 5, 1-1-1, Higashi, Tsukuba, Ibaraki 305-8565, Japan

[3] Japan Science and Technology Agency (JST), Honcho 4-1-8, Kawaguchi 332-0012, Japan

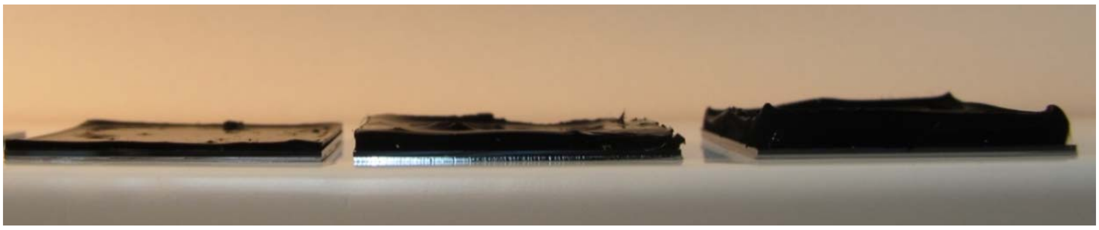

Figure S1. Photograph of SWCNT forest with different heights with Si substrate. From left to right: Forest with 350, 700, and 1500 μm height.

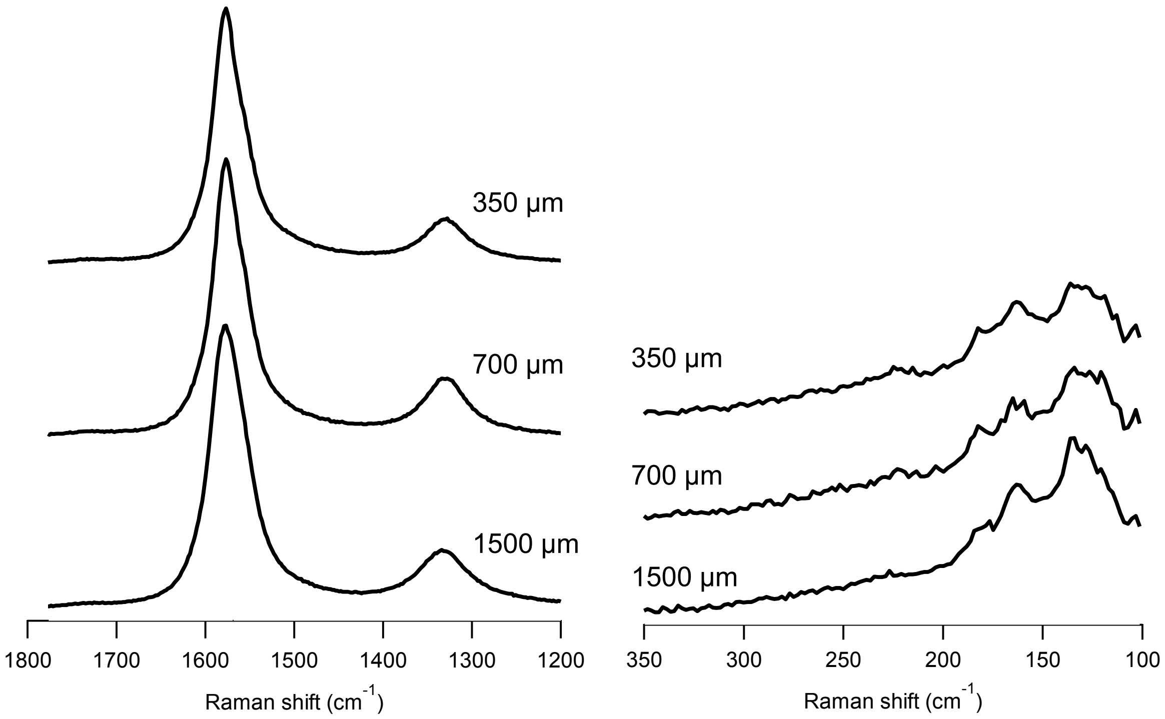

Figure S2. Raman spectra of SWCNT forest with different heights (excitation wavelength: 532 nm).
